# The dissipation of thiamethoxam and its main metabolite clothianidin during strawberry growth and jam-making process

**DOI:** 10.1038/s41598-018-33334-w

**Published:** 2018-10-15

**Authors:** Na Liu, Xinglu Pan, Qingxi Yang, Mingshan Ji, Zhihong Zhang

**Affiliations:** 1 Liaoning Key Laboratory of Strawberry Breeding and Cultivation, College of Horticulture, Shenyang Agricultural University, Shenyang, 110866 P. R. China; 2 State Key Laboratory for Biology of Plant Diseases and Insect Pests, Institute of Plant Protection, Chinese Academy of Agricultural Sciences, Beijing, 100193 P. R. China; 3 College of Plant Protection, Shenyang Agricultural University, Shenyang, 110866 P. R. China

## Abstract

Few studies focused on the residue of thiamethoxam and its metabolite clothianidin on strawberry where it is widely used, despite this is essential to assess the potential food risk of thiamethoxam and its main metabolite clothianidin. In this study, the dissipation of thiamethoxam and its metabolite clothianidin during strawberry growth and jam-making process were assessed. The strawberry was sprayed with thiamethoxam based on the field application to investigate the dissipation of thiamethoxam as well as clothianidin formation. The half-life of thiamethoxam in strawberry was 9.0 days and the concentration of clothianidin in strawberry gradually increased from 0.55 to 11 μg/kg within 30 days. In addition, the amount of thiamethoxam decreased by 51.7% and clothianidin decreased by 40.2% during the homogenization process. The processing factor values of whole processing all less than 1 except simmering. This results from this study will not only help to understand the dissipation kinetics of thiamethoxam and clothianidin in the strawberry, but also facilitate to make more accurate risk assessments of them during strawberry jam making process.

## Introduction

Strawberry (*Fragaria* × *ananassa Duch*) is a popular raw material for the food industry^1^. Especially strawberry jam and strawberry juice are popular for consumers. Hence, people are paying increasingly attention to food safety in strawberry products, especially pesticide residues^2^. However, few studies have focused on the concentration change during the processing. There are also no MRLs for the related processed commodities. Previous reports have indicated that processing techniques would reduce or increase the pesticide residues^2–4^. It was reported that peeling process caused the loss of 63.5% of chlorpyrifos and 53.3% of 3,5,6-trichloro-2-pyridinol in tomatoes^2^. Kong *et al*. (2012) found that the pesticide residues of chlorpyrifos, tebuconazole, acetamiprid and carbendazim were significantly reduced in the edible part of the apple during peeling and coring process^3^. Nevertheless, the concentration of fenthion sulfoxide was found to increase with the increasing of water addition in the olive oil extraction process^4^. Therefore, it is necessary to evaluate the pesticide residue during strawberry jam processing.

Thiamethoxam (*EZ*)-3-(2-chloro-1,3-thiazol-5-ylmethyl)-5-methyl-1,3,5-oxadiazinan-4-ylidene(nitro) amine, is a neonicotinoid insecticide actively against a broad range of insects in strawberry^5^. The previous report indicated that thiamethoxam labelled either in the 2-position of the thiazole moiety or on the carbon of the guanidine moiety (4-oxadiazine label) was used in the metabolism and environmental fate studies^5^. Thiamethoxam is transformed to clothianidin in soils, insects and plants, so the part of the insecticidal activity of thiamethoxam was also associated with its metabolite clothianidin^6^. However, the results showed that thiamethoxam and clothianidin would cause rats to release more dopamine *in vivo* striatum and it seems to be dose-dependent^7^. In addition, average residue levels of thiamethoxam in water systems have increased over the past 15 years, which reflected the worldwide trend in usage of this compound^8^. The no observed effect level from short term dietary test indicated that clothianidin was high toxic to rat^9^, and thiamethoxam would cause increased incidence of liver cell adenoma and adenocarcinoma in mouse^10^. Thus, it is of great significance to evaluate the behavior of thiamethoxam and its metabolite clothianidin during strawberry growth and jam-making process which will be important to make more accurate food safety assessment.

The objectives of the present work were to: (1) evaluate the dissipation of thiamethoxam and formation of its main metabolite clothianidin, (2) investigate the residual of thiamethoxam and clothianidin during strawberry jam making process, and (3) provide the information of thiamethoxam and clothianidin regarding PFs in strawberry jam making process. The results from this study may provide more experimental data in evaluating the jam safety contaminated by thiamethoxam and clothianidin.

## Results

### Recoveries of thiamethoxam and clothianidin in strawberry

No interference was found at the retention time for both compounds. The recoveries and RSDs of each compound were measured by spiking the blank samples with three different concentrations (i.e., 5, 50 and 500 μg/kg for thiamethoxam, 1, 10 and 100 μg/kg for clothianidin) and performing quintuplicate analysis (Table 1). All the recoveries were determined from the analysis of the target compounds in strawberry wine. As Table 1 showed, satisfactory mean recovery values (89.5–102.1%) and precisions were obtained; all experiments RSD values below 6.3% at the three fortified concentration levels. In general, the intra-day (n = 5) and inter-day RSDs (n = 15) for the proposed method ranged from 3.4–6.2% and 4.8–6.3%, respectively.

**Table 1:** Accuracy and precision of the proposed method in the studied matrices at three spiked levels^a^.

Compound	Matrix	Spiked level (µg/kg)	Intra-day (*n* = 5)						Inter-day (*n* = 15) RSD_R_ (%)
			Day 1		Day 2		Day 3		
			Mean recoveries (%)	RSD_r_ (%)	Mean recoveries (%)	RSD_r_ (%)	Mean recoveries (%)	RSD_r_ (%)	
Thiamethoxam	strawberry	5	93.1	3.4	96.0	3.8	89.5	3.6	5.1
		50	89.3	4.5	92.8	4.5	98.8	5.1	5.6
		500	88.6	3.9	95.1	3.9	92.4	4.1	4.8
Clothianidin		1	94.2	4.7	95.1	4.1	102.1	6.2	6.3
		10	93.5	5.1	101.2	3.6	97.8	4.2	5.1
		100	96.1	4.0	98.2	4.8	93.1	5.7	6.1

^a^RSD_r_ intra-day, the relative standard deviations for repeatability (n = 5); RSD_R_ inter-day, the relative standard deviations for reproducibility (n = 15).

The limit of quantifications (LOQs) of thiamethoxam and clothianidin were estimated at the lowest spiked concentration. In this work, the LOQs were estimated to be 5 and 1 μg/kg for thiamethoxam and clothianidin, respectively.

### Degradation of thiamethoxam and its metabolite clothianidin during strawberry growth

**Figure 1:**
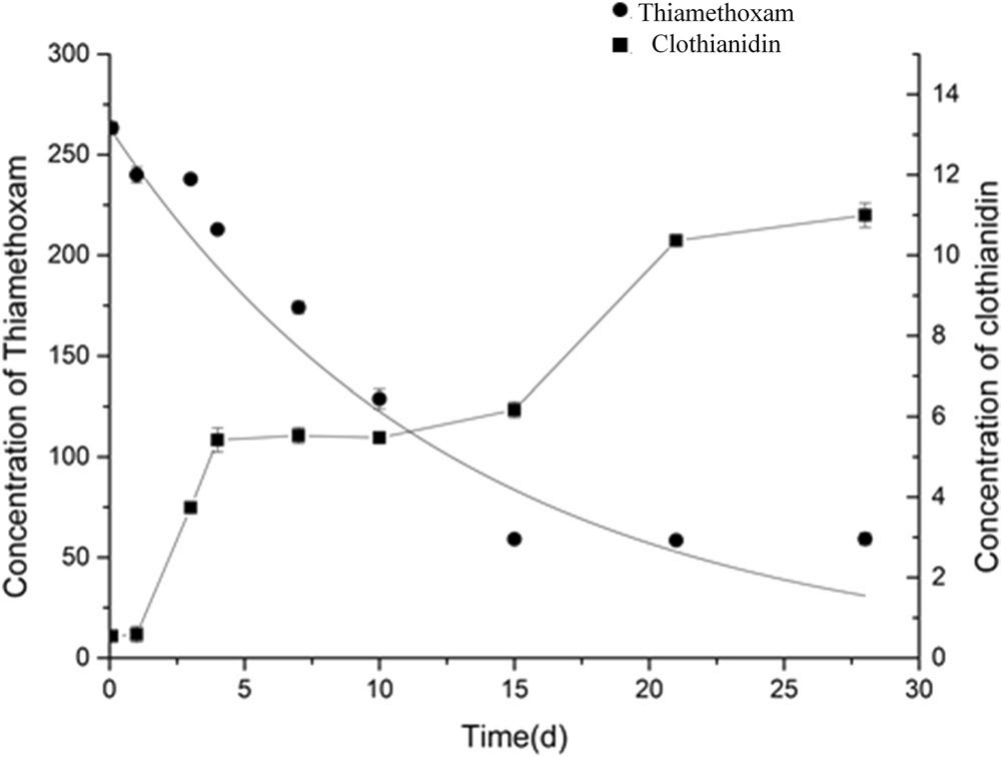
Changes in the concentrations of thiamethoxam and clothianidin in field strawberry.

To determine the dissipation of thiamethoxam and its metabolite clothianidin, samples were collected from the field plots after the application of thiamethoxam. The concentration changes of thiamethoxam and clothianidin in strawberry samples were profiled (Fig. 1). The results showed that thiamethoxam concentration decreased gradually with time elapse, while clothianidin concentration gradually increased. The dissipation rate of thiamethoxam in strawberry was fitted to first-order kinetics (R^2^ = 0.94549). The dissipation equation of thiamethoxam was *C*_*t*_ = 263.*3249e*^−0.*07694t*^, and the half-life of thiamethoxam in strawberry was 9.0 days. The concentration of clothianidin increased from 0.55 to 11.0 μg/kg in strawberry during 30 days of growth.

### Distribution of thiamethoxam and its metabolite clothianidin in strawberry after processing

**Figure 2:**
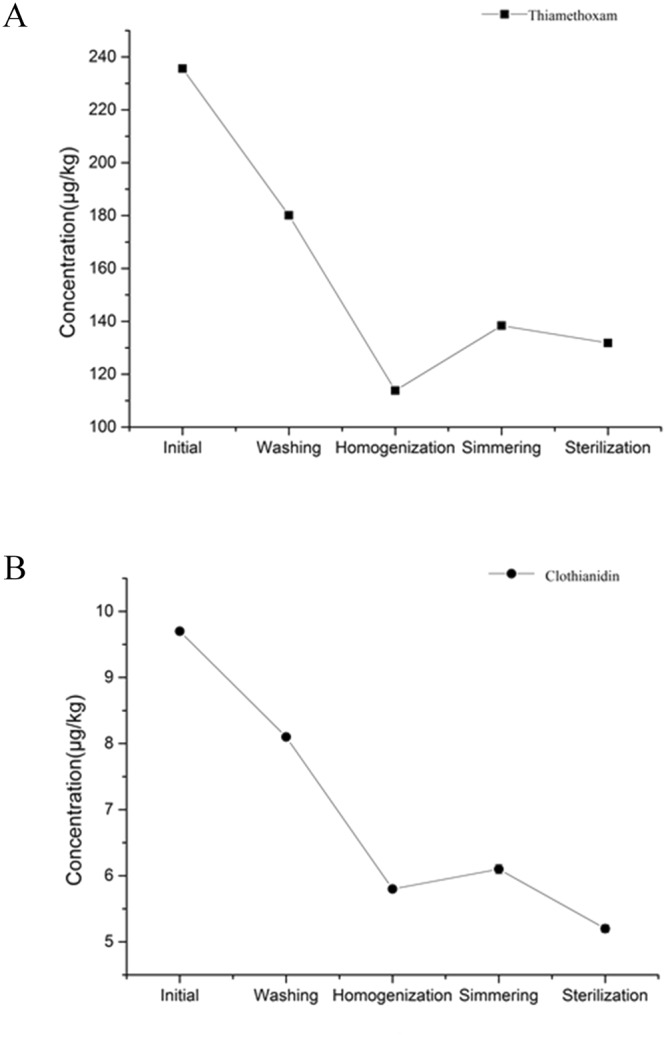
Trend of thiamethoxam and clothianidin content of canned strawberry jam by home processing.

A processing study was performed to evaluate the effect of technological steps on the residues of thiamethoxam and its metabolite clothianidin in strawberry. The concentrations of thiamethoxam and clothianidin during strawberry jam indicated that 4.0% of thiamethoxam was transformed to clothianidin in raw strawberry (Table 2). And the mean loss of thiamethoxam was 23.5%, and clothianidin was reduced by 16.5% (Table 2). The results showed that the amount of residues decreased by 51.7% for thiamethoxam and by 40.2% for clothianidin after the homogenization process. The residues of thiamethoxam and clothianidin in strawberry jam were more than those in strawberry puree (Fig. 2). In the sterilization, the results showed that the thiamethoxam and its metabolite clothianidin residues in canned strawberry jam were lower than those in unpasteurized strawberry jam.

**Table 2:** Amount of Thiamethoxam and Clothianidin residues (μg kg^−1^) recovered from strawberry samples after various home processes (mean ± SD).

Sample	Thiamethoxam (mean ± SD)	Clothianidin (mean ± SD)
raw strawberry	235.6^a^ ± 0.03	9.7^a^ ± 0.005
Washed strawberry	180.1^b^ ± 0.56	8.1^b^ ± 0.006
strawberry puree	113.8^d^ ± 0.05	5.8^e^ ± 0.0006
strawberry juice	141.6^c^ ± 0.07	6.9^c^ ± 0.0003
strawberry paste	138.4^e^ ± 0.05	6.1^d^ ± 0.007
Canned strawberry paste	131.8^f^ ± 0.04	5.2^f^ ± 0.006

^a–f^Values with the different letters are significantly different (*p* < 0.05).

### Processing factors

The processing factor (PF: the ratio of residue levels in processed commodities to those in the raw agricultural commodity) is a main parameter used in the acute dietary exposure assessment^11^. The calculated PF values for thiamethoxam and clothianidin after processing were all less than 1 except simmering (Table 3), indicating that the residue reduction in the processed commodity. The lowest PFs value was observed in the homogenization procedure.

**Table 3:** PFs for different processing type.

Processing type	PFs (Thiamethoxam)	PFs (Clothianidin)
Washing	0.76	0.83
Homogenization	0.63	0.72
Simmering	1.21	1.05
Sterilization	0.95	0.85

## Discussion

### Distribution of thiamethoxam and clothianidin in strawberry during processing

The maximum residue appeared in the unwashed strawberry. This maybe because the pesticide first attached on the strawberry surface. Washing is indispensable procedure to reduce pesticide residues which is specific to raw fruits. Washing as a process is prevalent in most households since it can be done with easily available tap water and also with solutions formulated from chemicals readily available in a household kitchen^12^.

The results difference in the residue reduction during the homogenization process between thiamethoxam (51.7%) and clothianidin (40.2%) may be related to the solubility of thiamethoxam and clothianidin in water. The solubility of thiamethoxam in water at 20 °C was 4100 mg/L and the solubility of clothianidin in water at 20 °C was 340 mg/L^9,10^. Due to the high water solubility, thiamethoxam was more easily to move into the juice and then was removed through the filtration process. Liu *et al*. (2014) found that thiophanate-methyl and carbendazim were difficult to transport into the internal parts of tomato juice and tomato seeds because of their low water solubility^13^.

About a half of water in the fruit was evaporated during simmering^14^. The residues of thiamethoxam and clothianidin in strawberry jam were more than those in strawberry puree. The result may be because the pesticides were concentrated as the water evaporated from the strawberry puree^15^.

In the sterilization, the results showed that the thiamethoxam and its metabolite clothianidin residues in canned strawberry jam were lower than those in unpasteurized strawberry jam. Similar findings were obtained by Li *et al*. (2011), who found that sterilization eliminated the cypermethrin and prochloraz residues^16^. The results indicate that the processing steps can obviously reduce thiamethoxam and clothianidin residues and thus corresponding risks to human intake.

### Processing factors

The “Joint Meeting on Pesticide Residues” (JMPR) stipulated that the aim of food processing studies on residues is relate the levels of residue in processed products to that in the raw agricultural commodity and to calculate processing factors (PFs) from trials^17^. The PF was the ratio of residue levels in processed commodities to those in the raw agricultural commodity^18^. If the PF values less than 1 (i.e., reduction factor) indicated a reduction of residues in a processed commodity, whereas the values more than 1 (i.e., concentration factor) demonstrated concentration effects from the processing procedures^19^.

The PF value of simmering was more than 1 which maybe because the process of simmering would concentrate the thiamethoxam and clothianidin residue as a result of the water evaporation. The lowest PFs value was observed in the homogenization procedure. The results demonstrated that homogenization played the most important role in removing residues among all the processing steps. Many factors could affect the removal of residue levels, for instance, physicochemical property of the pesticide, processing mode, and so on^18^. Hence, taking more concern on the change of the pesticide residues during processing is particularly necessary for the further food safety.

## Conclusions

In this study, the fate of thiamethoxam and its metabolite clothianidin during strawberry growth and jam-making process was investigated. As the concentration of thiamethoxam decreased gradually with time elapse, the concentration of metabolite clothianidin increased. The half-life of thiamethoxam in strawberry was 9.0 days. Different processing procedures could affect the reduction of thiamethoxam and clothianidin residues. The results showed that the residue reductions in the whole processing with the PFs were less than 1 except simmering. Homogenization played the most important role in removing residues of thiamethoxam and clothianidin among the processing steps. The results might provide more accurate risk assessments of thiamethoxam and clothianidin in jam-making process.

## Materials and Methods

### Chemicals and materials

The analytical standard thiamethoxam (98.2% purity) and its metabolite clothianidin (99.0% purity) obtained from the China Standard Material Center (Beijing, China). Commercial 25% thiamethoxam water dispersible granule (WG) was obtained from Swiss Syngenta Crop Protection co., LTD (Shanghai, China). Acetonitrile, anhydrous magnesium sulfate and sodium chloride for pesticide residue analysis were of analytical grade and purchased from Beijing Chemical and Reagent (Beijing, China). Acetonitrile and methanol (chromatography grade) were obtained from Fisher Scientific (Shanghai, China). Primary secondary amine (PSA, 40 μm) and graphitized carbon black (GCB) purchased from Agela Technologies (Tianjin, China). Ultra-pure water was obtained from a Milli-Q system (Bedford, MA, USA).

Standard stock solutions of thiamethoxam (100 mg/L) and clothianidin (100 mg/L) were prepared in acetonitrile (Chromatography grade). The standard solutions required for construction of a calibration graph (0.005, 0.01, 0.05, 0.1, 0.5 and 1 mg/L for thiamethoxam; 0.001, 0.005, 0.01, 0.05, 0.1 and 0.5 mg/L for clothianidin) were prepared from stock solutions by serial dilution with acetonitrile (Chromatography grade). Correspondingly, matrix-matched standard solutions were obtained at a series of concentrations (0.005, 0.01, 0.05, 0.1, 0.5 and 1 mg/L for thiamethoxam; 0.001, 0.005, 0.01, 0.05, 0.1 and 0.5 mg/L for clothianidin) by adding blank strawberry sample extracts to each serially diluted standard solution. All solutions were stored in a refrigerator in the dark at 4 °C and the working standard solutions underwent no degradation for 3 months.

### Instrumentation

Chromatographic separation was carried out with an Agilent 1290 high-performance liquid chromatography (HPLC) which equipped with an Eclipse Plus C18 column (2.1 mm × 50 mm, 1.8 μm particle size) (Milford, MA, USA). Gradient UPLC elution was performed with acetonitrile (Chromatography grade) as mobile phase A and 0.2% (*v/v*) formic acid in water as mobile phase B. The flow rate of 0.5 mL/min with an gradient elution of 0 min 10% A phase, 1.0 min 25% A phase, 1.5 min 95% A phase, 4.0 min 95% A phase, 5 min 10% A phase. Separation was achieved in 4.0 min. The column was kept at 45 °C in order to decrease viscosity and the temperature in the sample manager was set at 5 °C and the sample volume injected was 1 µL.

**Figure 3:**
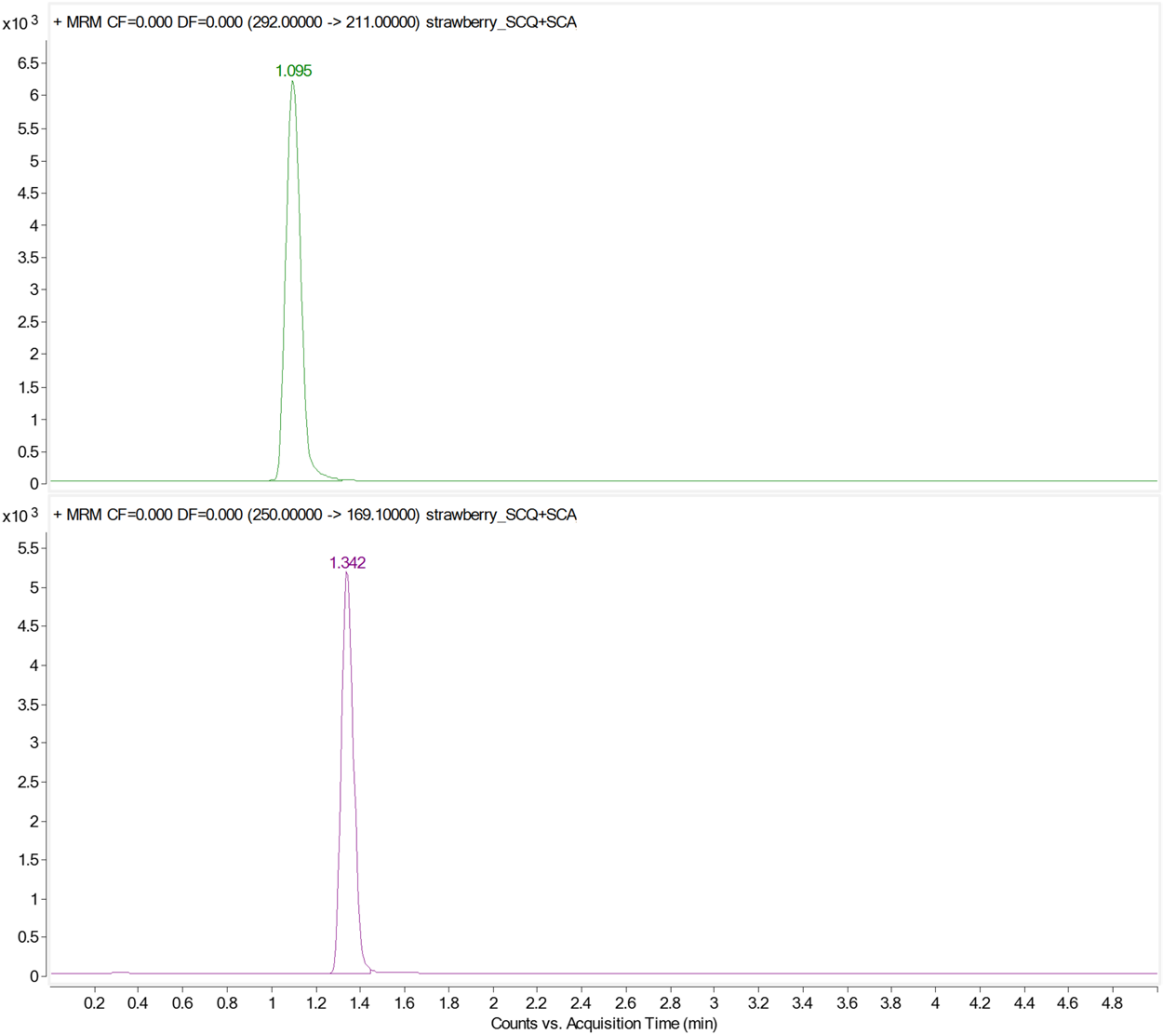
Typical UPLC-MS/MS chromatograms of a standard of Thiamethoxam and Clothianidin.

Detection of thiamethoxam and clothianidin were conducted on a triple-quadrupole mass spectrometry (QQQ, Agilent Technologies), equipped with an Agilent Jet Steam ESI source was used to quantify the target compounds. Nitrogen was used for both nebulizer and collision gas. MS/MS detection was performed in positive ion mode. The monitoring conditions, multiple reaction mode (MRM), were optimized for target compound, with a dwell time of 90 ms, respectively. The conditions were typical as follows: The nebulizer gas was 99.95% nitrogen with an output pressure of 100 psi, obtained from nitrogen generator, and the collision was 99.999% nitrogen with a pressure of 0.2 MPa. The gas and sheath gas temperature were 325 and 350 °C with the flow rate 8.0 and 10 L/min, respectively. The capillary voltage was set at 4.5 kV and the nozzle voltage was 500 V. A dwell time of 90 ms per ion pair was used to maintain the high sensitivity of the analysis, and a number of data points across the chromatographic peak were required. The typical conditions were as follows: the fragmentor voltage of thiamethoxam was 90 V; m/z 292 was selected as the precursor ion for thiamethoxam, m/z 181 was selected for the product quantitative ion, and m/z 211 was selected for the qualitative ion when the collision energy was set to 20 and 10 V. The fragmentor voltage of clothianidin was 90 V; m/z 250 was selected as the precursor ion for clothianidin, m/z 132 was selected for the product quantitative ion, and m/z 169.1 was selected for the qualitative ion when the collision energy was set to 10 and12 V. And the cell accelerator voltage of thiamethoxam and clothianidin were both 4 V. Under the conditions, the retention times of thiamethoxam and clothianidin were approximately 1.1 and 1.3 min, respectively (Fig. 3). These settings were utilized for all subsequent studies. The MassHunter Workstation Software Version B.08.00 (Agilent Technologies, USA) was used to process data obtained from different matrices and from calibration standards.

### Extraction and purification procedure

A portion of 10 g homogenized samples were weighed into a 50 mL PTFE centrifuge tube. Then, 10 mL acetonitrile were added, and the mixture was placed on a Geno/Grinder mechanical shaker (SPEX Sample Prep, USA) for 3 min at 1200 strokes min^−1^. A total of 4 g of anhydrous MgSO_4_ and 1 g of NaCl was added and vortexed with an XW-80A Vortex (Kirin Medical Instrument, China) at full speed for 1 min and then the tube were centrifuged with a TG16-WS centrifuge (Xiangyi Centrifuge Machines, China) for 5 min at relative centrifugal force (RCF) 2077 × *g*. Then, 1.5 mL supernatant was transferred to a single-use centrifuge tube containing 30 mg PSA sorbents, 20 mg GCB sorbents and 150 mg anhydrous MgSO_4_, and then vortexed at full speed for 1 min and centrifuged briefly. After that, 1 mL of upper layer was filtered with 0.22 μm Nylon syringe filters (15 mm diameter, Agela Technologies, China) for UPLC-MS/MS analysis.

### Sample preparations

**Figure 4:**
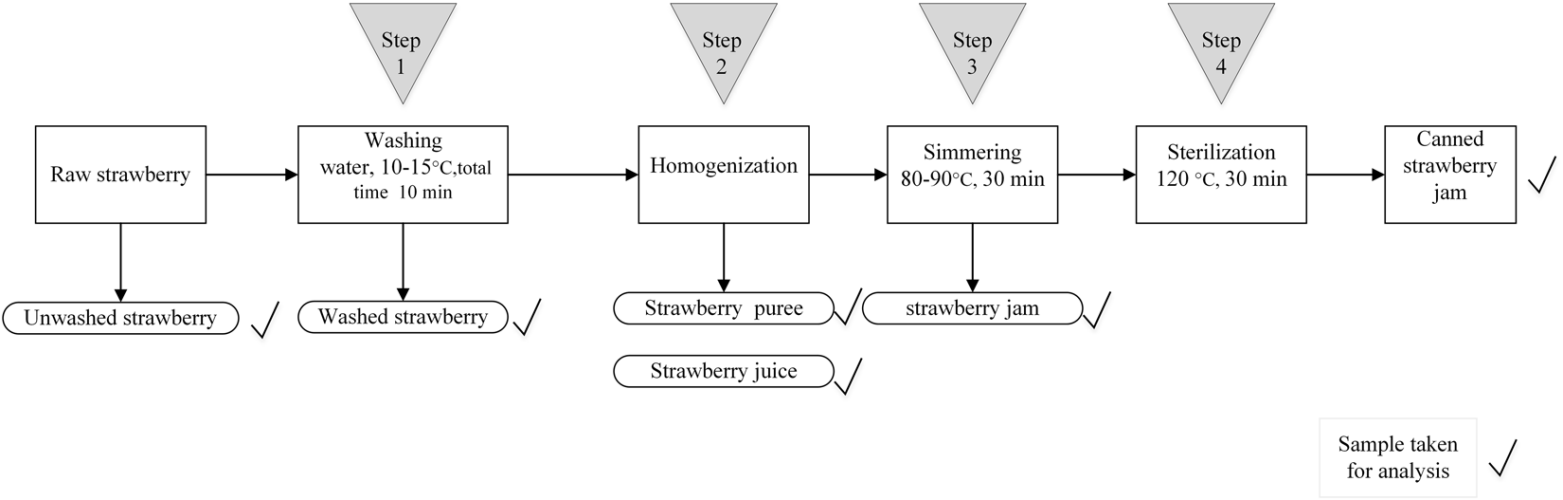
Scheme for traditional canned strawberry jam processing used in this study and sampling points.

In general, the production procedures of canned strawberry paste include four steps, washing (remove the sepals), homogenization, simmering, and sterilization, as shown in Fig. 4. The first step was washing. And then the washed strawberry samples were homogenized using a blender to preserve its taste. The next process was simmering. Put the strawberry puree into saucepan and heat for half an hour. This procedure was applied to remove excess water from strawberry puree. Finally, the Strawberry jam was sterilized at 120 °C for 30 min and then canned. The samples (unwashed strawberry, washed strawberry, puree, juice, paste, and canned strawberry paste) in different processing steps were collected to determine and investigate the variation of pesticide residue during the processing procedure.

### Recovery assay

The recovery test was carried out to investigate the accuracy and precision of the method. Five replicates of the spiked samples at different levels (i.e., 5, 50 and 500 μg/kg for thiamethoxam, 1, 10 and 100 μg/kg for clothianidin) for strawberry were prepared on three different days. The precision in these conditions for repeatability, expressed as the relative standard deviation (RSD), was determined by the intra- and inter-day assays. Prior to the extraction step, the spiked samples were allowed to settle for 30 min at room temperature to make sure the target compound penetrate into the matrices evenly. Then, the extraction and purification procedures were conducted according to the aforementioned method. The recoveries obtained with the extracted spiked samples were compared with that of the matrix-matched calibration solutions. Calibration curves in the matrix, which were prepared by using this method, automatically corrected the data for analytical recovery.

### Data analysis

The dissipation kinetics of thiamethoxam and its metabolite clothianidin during strawberry growth were estimated using the first-order kinetics equation. The half-life of each compound was measured using the following equations^20^:

$$C={C}_{0}{e}^{-kt},\,{T}_{1/2}=ln\,2/k.$$

where C_0_ and C indicated the concentration of the compound at initial time and time t. *k* is the dissipation rate constant.

The data were analyzed using OriginPro v.8.0 (OriginLab, Northampton, MA, USA). All experiments were performed at least three times. The results of the concentrations are expressed as the means ± SD from the independent experiments, and one-way analysis of variance with Duncan’s multiple range test (SPSS Inc., Chicago, IL, USA) was used to compare the differences among means.

### Field trials

The trials were conducted in an experimental greenhouse located in Shenyang, China. And thiamethoxam and clothianidin were not applied in the trial plots in the past three years. The experimental field was divided into seven plots. Each plot was 30 m^2^. The experiment was designed with three replicated plots for each treatment and the seventh plot as a control. To ensure sufficient pesticide primary deposit for the following processing study, 25% thiamethoxam WG was applied under greenhouse condition at dosage of 1125 g/hectare (5 times of recommended higher dosage) during strawberry maturation^21^. And 25% thiamethoxam WG was applied at dosage of 337.5 g/hectare (1.5 times of recommended higher dosage) during the strawberries grow to half size for dissipation dynamics study. The pesticide was sprayed with an LP-605 (Agrolex, Singapore) manual sprayer on March 15 for dissipation dynamics study, and March 28, 2017 for processing study. Strawberry samples were sampled after 2 h, 1, 3, 5, 7, 10, 14, 21, 28 and 35 days for dissipation dynamics study. And two kilograms strawberry samples per plot were sampled at 3 days after the treatment for processing study. All plant samples were placed in polyethylene bags and transported to the laboratory in the same day. The samples were processed within 24 h after being collected from the field. All the processed samples were stored at −20 °C until further analysis.
